# Compassionate use of a novel β-lactam enhancer-based investigational antibiotic cefepime/zidebactam (WCK 5222) for the treatment of extensively-drug-resistant NDM-expressing *Pseudomonas aeruginosa* infection in an intra-abdominal infection-induced sepsis patient: a case report

**DOI:** 10.1186/s12941-023-00606-x

**Published:** 2023-07-05

**Authors:** Dilip Dubey, Manish Roy, Tajamul H. Shah, Noor Bano, Vidushi Kulshrestha, Sandeep Mitra, Pushpender Sangwan, Madhulika Dubey, Ali Imran, Bhawna Jain, Aravind Velmurugan, Yamuna Devi Bakthavatchalam, Balaji Veeraraghavan

**Affiliations:** 1 Institute of Critical Care medicine, Medanta, Lucknow India; 2 Department of Pulmonary Medicine, Sher-I-Kashmir Institute of Medical Sciences, Soura, Srinagar, India; 3 Department of Critical Care Medicine, ERA’s Lucknow Medical College and Hospital, Lucknow, India; 4 Department of Microbiology, Medanta, Lucknow India; 5 Department of Clinical Microbiology, Christian Medical College and Hospital, Vellore, India

**Keywords:** Cefepime/zidebactam, β-lactam-enhancer, Pseudomonas, New-Delhi metallo-β-lactamase, Extensively-drug-resistant

## Abstract

Infections in critically-ill patients caused by extensively-drug-resistant (XDR)-*Pseudomonas aeruginosa* are challenging to manage due to paucity of effective treatment options. Cefepime/zidebactam, which is currently in global Phase 3 clinical development (Clinical Trials Identifier: NCT04979806, registered on July 28, 2021) is a novel mechanism of action based β-lactam/ β-lactam-enhancer combination with a promising activity against a broad-range of Gram-negative pathogens including XDR *P. aeruginosa*. We present a case report of an intra-abdominal infection-induced sepsis patient infected with XDR *P. aeruginosa* and successfully treated with cefepime/zidebactam under compassionate use. The 50 year old female patient with past-history of bariatric surgery and recent elective abdominoplasty and liposuction developed secondary pneumonia and failed a prolonged course of polymyxins. The organism repeatedly isolated from the patient was a New-Delhi metallo β-lactamase-producing XDR *P. aeruginosa* resistant to ceftazidime/avibactam, imipenem/relebactam and ceftolozane/tazobactam, susceptible only to cefepime/zidebactam. As polymyxins failed to rescue the patient, cefepime/zidebactam was administered under compassionate grounds leading to discharge of patient in stable condition. The present case highlights the prevailing precarious scenario of antimicrobial resistance and the need for novel antibiotics to tackle infections caused by XDR phenotype pathogens.

**Supplementary Information:**

The online version contains supplementary material available at 10.1186/s12941-023-00606-x.

## Main text

Extensively-drug-resistant (XDR) *Pseudomonas aeruginosa* (non-susceptible to at least one agent in all but one or two anti-pseudomonal antibiotic classes) are usually related to certain sequence-types (ST) that have disseminated across the world and termed “high-risk” clones [1] The worldwide top 10 *P. aeruginosa* high-risk clones include ST235, ST111, ST233, ST244, ST357, ST308, ST175, ST277, ST654 and ST298 [2]. Infections of XDR *P. aeruginosa* are associated with higher mortality rate, prolonged hospitalization and an increased treatment cost compared with infections caused by antibiotic-susceptible *P. aeruginosa* [3]. In recent years, few new anti-pseudomonal antibiotics (ceftolozane/tazobactam, ceftazidime/avibactam, imipenem/relebactam and cefiderocol) have been introduced. While they are active against certain XDR isolates, yet are riddled with spectrum gaps. For instance, against metallo-β-lactamase (MBL)-expressing organisms, none of the new β-lactam/β-lactamase inhibitors exhibit any meaningful activity and cefiderocol demonstrates elevated MICs against such resistotype [4, 5].

Cefepime/zidebactam (WCK 5222) is a novel β-lactam enhancer mechanism based combination currently being studied in a global Phase 3 trial in adult patients with complicated urinary tract infection or acute pyelonephritis (ClinicalTrials.gov identifier: NCT04979806). Zidebactam is distinguished from newer β-lactamase inhibitors such as avibactam and taniborbactam by means of an additional function; selective and high-affinity binding to penicillin-binding protein (PBP) 2. When combined with cefepime that targets PBP3, zidebactam synergistically enhances the bactericidal activity of cefepime, thus functioning as a “β-lactam-enhancer” [6, 7]. In vitro and in vivo studies conducted on cefepime/zidebactam have established its broad-spectrum activity against Gram-negative organisms that include both serine-carbapenemases and MBL- expressing isolates [8–11].

**Fig. 1:**
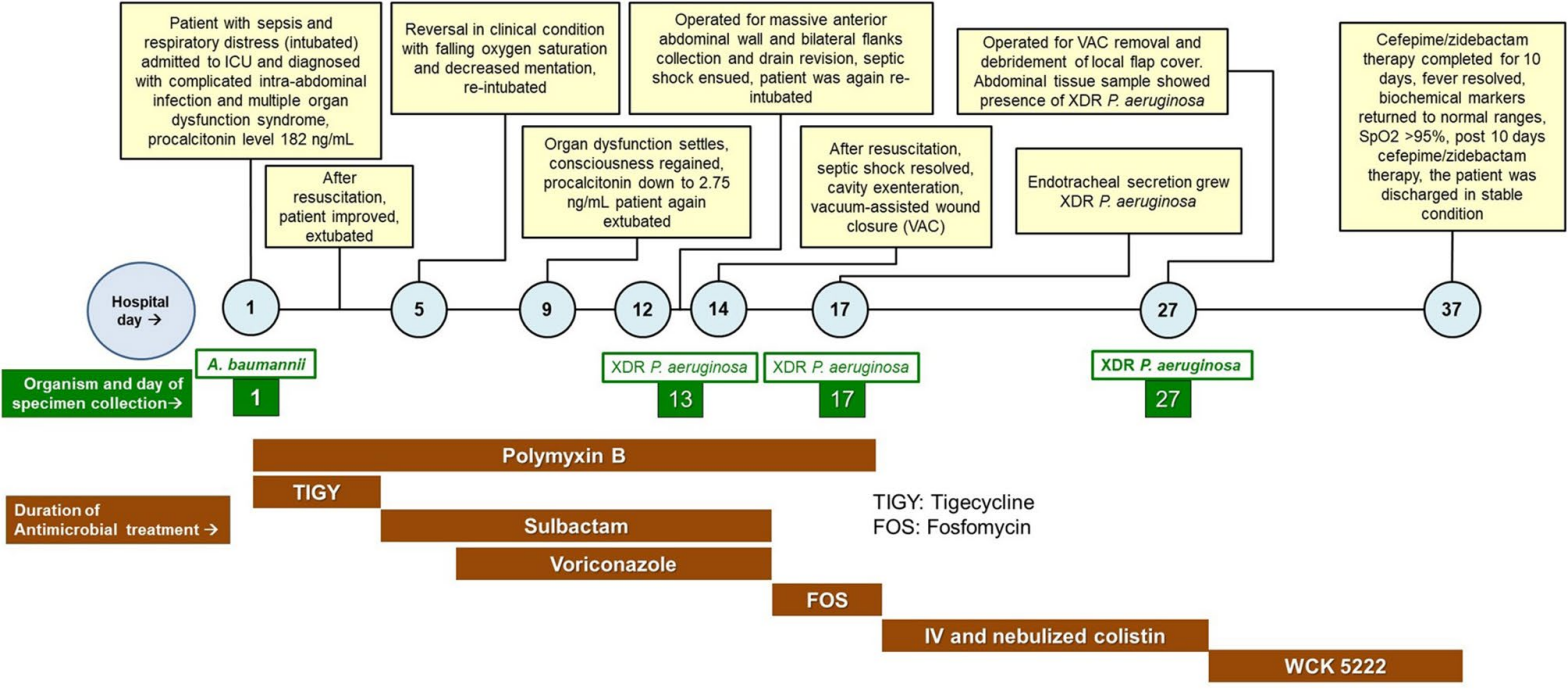
Events during the hospital stay of the patient

During the month of August, 2022, a critically-ill patient with a complex XDR *P. aeruginosa* infection was admitted to our tertiary care hospital. With last line antibiotics failing to eradicate the pathogen, the patient was successfully treated with cefepime/zidebactam under the compassionate use. This is the first case of compassionate use of cefepime/zidebactam and the clinical events surrounding the patient from the day of hospitalization to discharge are described below (Fig. 1).

## Case report

A 50-year-old woman from Nepal with a past-history of bariatric surgery (2013) was presented to a speciality hospital in Lucknow, India for an elective abdominoplasty and liposuction. Post-surgery, she developed intra-abdominal sepsis and septic shock with features of severe hypoxemic respiratory failure. She required invasive mechanical ventilation and was subsequently shifted to the critical care unit of Medanta Hospital, Lucknow, India for further management. On admission, the patient was diagnosed with multi-organ failure with high SOFA (Sequential Organ Failure Assessment) score and serum procalcitonin level was 182 ng/mL. In view of suspected Gram-negative-implicated sepsis, an empiric antibiotic regimen consisting of polymyxin B and tigecycline was initiated. Over the next 48–72 h, patient’s condition improved with a significant reduction in the requirement of vasopressors and a drop in the serum procalcitonin level to 32.8 ng/mL. Subsequently, she was extubated after successful spontaneous breathing trial. However, by hospital-day 4, patient experienced respiratory distress and deteriorating clinical condition warranting reintubation, which was performed on hospital-day 5.

Meanwhile, the endotracheal aspirate collected on day 1 showed a growth of *Acinetobacter baumannii* with susceptibility to polymyxins only (EUCAST criteria). Noting this, tigecycline was discontinued and high-dose sulbactam was added with in vitro-active polymyxin B on day 4. Moreover, in view of persistent fever and vasopressor requirement, the antimicrobial regime was further augmented by addition of anti-fungal, voriconazole on day 6 [12].

The patient remained febrile without clinical improvement and continued to be under mechanical ventilation. The ensuing endotracheal sample (day 13) didn’t show *A. baumannii*, rather a new organism, *P. aeruginosa* (ISOLATE 1) with susceptibility only to polymyxins (EUCAST criteria). The isolate was resistant to ceftazidime/avibactam, ceftolozane/tazobactam, and imipenem/relebactam (Table 1). Based on the susceptibility to fosfomycin as reported by VITEK® 2 (later turned to be resistant in reference MIC method), intravenous fosfomycin was added to on-going polymyxin B while sulbactam and voriconazole were discontinued (day 15). On day 17, a 3rd endotracheal sample was collected which once more revealed the presence of *P. aeruginosa* (ISOLATE 2) with susceptibility to polymyxins (EUCAST criteria) but resistant to fosfomycin (VITEK® 2 & reference MIC > 128 mg/L). With no improvement in the clinical condition of the patient, fosfomycin and polymyxin B were withdrawn, replaced with intravenous and nebulized colistin (day 19).

**Table 1:** Antibiotic susceptibility of three XDR *P. aeruginosa* collected from the patient during the clinical course

Antibiotics		MICs (mg/L)			
		Endotracheal secretion samples			Abdominal tissue sample
		hospital day 13	hospital day 17		hospital day 27
		**ISOLATE 1**	**ISOLATE 2**		**ISOLATE 3**
Cefepime		> 64	> 64		> 64
Cefepime/zidebactam		16	16		16
Ceftazidime		> 64	> 64		> 64
Ceftazidime/avibactam		> 64	> 64		> 64
Imipenem		> 64	> 64		> 64
Imipenem/relebactam		> 64	> 64		> 64
Ceftolozane/tazobactam		> 64	> 64		> 64
Piperacillin/tazobactam		> 64	> 64		> 64
Meropenem		> 64	> 64		> 64
Amikacin		> 64	> 64		> 64
Levofloxacin		> 64	> 64		> 64
Fosfomycin		> 256	> 256		> 256
Colistin		0.25	0.25		0.25
Polymyxin B		0.12	0.25		0.25
Imipenem + EDTA		16	16		16
Ceftazidime/avibactam + EDTA		16	16		16

Cefepime/zidebactam MICs were determined in 1:1 ratio;

For all the β-lactamase inhibitor based combination, inhibitor was at fixed 4 mg/L

EDTA: Ethylene diamine tetraacetic acid at fixed 200 mg/L

For fosfomycin, agar MIC method was employed by supplementing the medium with glucose-6-phosphate at 25 mg/L

## Compassionate use of cefepime/zidebactam

Amidst the continued ICU stay of the patient with sepsis-multiple organ dysfunction syndromes (MODS), repeated recovery of an XDR *P. aeruginosa* and dwindling antibiotic choices, susceptibility of an investigational antibiotic cefepime/zidebactam was requested after obtaining requisite permissions from Drugs Controller General of India. Cefepime/zidebactam MIC was determined as per Clinical and Laboratory Standards Institute (CLSI M100 Ed 32 USA) guidelines and was noted as 16 mg/L, below its PK/PD breakpoint of ≤ 32 mg/L [8, 13]. With data supporting susceptibility, cefepime/zidebactam monotherapy was initiated on day 28 (replacing intravenous and nebulized colistin) under compassionate use, as per the manufacturer’s dosing instructions. Meanwhile, on day 27 (prior to initiation of cefepime/zidebactam), the abdominal tissue sample was submitted for microbiological examination, which showed the presence of *P. aeruginosa* (ISOLATE 3) with susceptibility profile identical to that of ISOLATE 2. To note, previous to cefepime/zidebactam administration, abdominal wound management was undertaken with debridement on day 13, 21 and 27. The vacuum-assisted closure of wound was performed on day 14.

Post administration of cefepime/zidebactam, the patient showed gradual improvement in the clinical condition and within three days of therapy, fever was resolved. With continuous administration of cefepime/zidebactam, a significant improvement in respiratory and hemodynamic parameters was noticed, and the patient was shifted to general ward for further care. As patient was out of intubation and with resolution of abdominal drain discharge, no specimen for bacterial culture could be collected. Meanwhile, to generate an evidence of clearance of pathogen, on day 7 of cefepime/zidebactam treatment, blood was collected which yielded no culture growth. After receiving 10 days of cefepime/zidebactam therapy with no reported adverse drug events, the patient was discharged in stable condition on hospital day 37. Two follow-up visits on 11 and 36 days after the discharge confirmed the complete recovery paving way for her return to the native country.

## Antimicrobial resistance profile of isolates

For all three *P. aeruginosa* isolates, MICs of carbapenems (> 64 mg/L) in the presence of EDTA was lowered by > 8 times, which phenotypically indicates the presence of MBL. This was confirmed by the detection of *bla*_NDM−1_ in whole genome sequence analysis (Supplementary Table 1). Importantly, all three isolates were clonally identical and belonged to an international high-risk clone of *P. aeruginosa*, ST357. In the single nucleotide polymorphism analysis, mutations in efflux proteins (e.g., MexB, MexC, MexEF), OprD, and β-lactam target (*ftsI* gene; PBP3), that are known to cause high level of β-lactam resistance were observed [14, 15] Further, the isolates showed presence of acquired genes and mutations linked with resistance to aminoglycosides, fluoroquinolones, and tetracyclines (Supplementary Table 1).

## In vitro time-kill activity

Time-kill studies were undertaken to assess the bactericidal activity of cefepime/zidebactam against *P. aeruginosa* isolated from the patient. Standalone cefepime was ineffective in restricting the growth of organisms even at higher concentrations. On the other hand, at 2x MIC, cefepime/zidebactam showed a time-dependent bactericidal action (~ 1.5-3-log_10_). Although colistin showed rapid killing initially (2 h), however a bacterial regrowth was observed at later time-points (6–8 h) suggesting selection and proliferations of tolerant population (supplementary Fig. 1).

## Discussion

In contrast to Enterobacterales, multi-drug resistance in *P. aeruginosa* is principally mediated through non-enzymatic resistance mechanisms such as down regulated porins or/and hyper-expression of efflux pumps. From therapeutic perspectives, for such resistotypes, newer anti-pseudomonal drugs show about 60–80% coverage [16, 17], however, they are ineffective against MBL-expressing *P. aeruginosa* commonly prevalent in Asia, particularly in India. These isolates are often co-resistant to aminoglycosides and fluoroquinolones leaving polymyxins as the only treatment option. However, in the present case, polymyxins were unable to eradicate the pathogen despite their prolonged use. This is not unexpected in view of their significant inter-patient PK variability and PK/PD insufficiency in body sites such as lung and intra-peritoneal fluid [18]. Moreover, the treatment of serious carbapenem-resistant Gram-negative infections with colistin-based therapies has been associated with 40% mortality and about 50% acute kidney injury. All three isolates were also resistant to intravenous fosfomycin (MIC > 256 mg/L). In this context, compassionate use of an investigational drug cefepime/zidebactam helped eradicate the pathogen leading to substantial improvement in the clinical condition of the patient. Moreover, no drug-linked adverse effects were noticed. Previous in vitro studies have shown promising activity of this combination against XDR *P. aeruginosa* including those producing MBLs [8]. Translational in vivo studies in neutropenic mice lung or thigh models also showed efficacy of cefepime/zidebactam against MBL-expressing *P. aeruginosa* at exposures mimicking human exposures [10, 19]. Thus, non-clinical studies support the potential of cefepime/zidebactam for the treatment of infections caused by XDR *P. aeruginosa*.

Albeit the favourable outcome in the end, the present case highlights the dearth of safe and effective antibiotics to deal with the infections caused by XDR *P. aeruginosa*. Importantly, given the epidemiological diversity in the resistance mechanisms, there is a pressing unmet need for the novel antibiotics that comprehensively addresses all the resistance mechanisms associated with XDR *P. aeruginosa*.

## Conclusion

Serious infections caused by XDR-phenotype Gram-negative organisms are associated with poor clinical outcome mainly due to non-availability of effective & safe antibiotics. Specifically, infections caused by MBL-expressing *P. aeruginosa* pose severe therapeutic challenges as none of the newer β-lactam/β-lactamase inhibitors are effective against such pathogens. Novel β-lactam enhancer mechanism based cefepime/zidebactam is being developed targeting XDR Gram-negatives including MBL- producers. In this present case, compassionate use of cefepime/zidebactam was opted as a salvage therapy as no other therapeutic options were effective.

## Electronic supplementary material

Below is the link to the electronic supplementary material.

Supplementary Material 1

Supplementary Material 2

## Data Availability

The whole genome sequence datasets generated and/or analysed during the current study are available on the NCBI Link: https://www.ncbi.nlm.nih.gov/nuccore?term=900707%5BBioProject%5D. ***Pseudomonas isolate no.  Accession number***: Isolate 1 (LRK01)     JAPHVT000000000.1. Isolate 2 (LRK02)     JAPHVU000000000.1. Isolate 3 (LRK03)     JAPHVV000000000.1.

## Abbreviations

XDR: Extensively-drug-resistant
SOFA: Sequential organ failure assessment
PBP: Penicillin binding protein
MIC: Minimum inhibitory concentration
MLST: Multi-locus sequence type
ST: Sequence type
MBL: Metallo-β-lactamase
MODS: Multiple organ dysfunction syndrome
CLSI: Clinical and Laboratory Standards Institute
