# Supplementary material for: Compassionate use of a novel β-lactam enhancer-based investigational antibiotic cefepime/zidebactam (WCK 5222) for the treatment of extensively-drug-resistant NDM-expressing Pseudomonas aeruginosa infection in an intra-abdominal infection-induced sepsis patient: a case report
Source: Ann Clin Microbiol Antimicrob. 2023 Jul 5;22:55. doi: 10.1186/s12941-023-00606-x (PMC10324185; doi:10.1186/s12941-023-00606-x)
Supplement: Supplementary file 1 — Supplementary Material 1 [file 12941_2023_606_MOESM1_ESM.docx]

**Supplementary Figure 1.** In vitro bactericidal activity of cefepime, cefepime/zidebactam, and colistin against XDR *P. aeruginosa* (Isolate 1, 2 and 3).

**Isolate 1 Isolate 2**

B

**Isolate 3**

Bacterial culture was grown in cation-adjusted Mueller-Hinton broth (MHB) in absence and presence of various concentrations of test antibiotics (shown in the graph). Samples were collected at various time-points (0, 2, 4, 6, and 8 h), and bacterial counts were determined by plating serial dilutions on tryptic soya agar. The extent of killing was estimated by comparing it with the initial inoculum seeded (~10^6^ cells/mL) and in comparison to culture in which no drug was added (growth control).
