# Supplementary material for: Compassionate use of a novel β-lactam enhancer-based investigational antibiotic cefepime/zidebactam (WCK 5222) for the treatment of extensively-drug-resistant NDM-expressing Pseudomonas aeruginosa infection in an intra-abdominal infection-induced sepsis patient: a case report
Source: Ann Clin Microbiol Antimicrob. 2023 Jul 5;22:55. doi: 10.1186/s12941-023-00606-x (PMC10324185; doi:10.1186/s12941-023-00606-x)
Supplement: Supplementary file 2 — Supplementary Material 2 [file 12941_2023_606_MOESM2_ESM.docx]

**Supplementary Table 1**. Summary of acquired and mutational resistance determinants in *P. aeruginosa* isolates*

| **MLST** | **β-lactamases** | **Acquired antibiotic resistance genes (antibiotic class)** | **Major single nucleotide polymorphisms** |
| --- | --- | --- | --- |
| ST-357 | PDC-3; VEB-1; NDM-1; OXA-50 | aac(6')-Il (aminoglycoside);  aph(6)-Id (aminoglycoside); aph(3')-IIb (aminoglycoside); aac(3)-Id (aminoglycoside); aph(3'')-Ib (aminoglycoside); rmtB (aminoglycoside); fosA (fosfomycin); tet(A) (tetracycline); tet(B) (tetracycline) | OprD: GS57EGR,V127L,EP185QG,V189T,E202Q,I210A,E230K,S240T,  N262T,T276A,A281G,K296Q,Q301E,R310E,A315G,Q424E  MexB: G957D,I186V  MexC: E218Q,A229E,A244T,H277R,S297A,A345T  MexD: T87S,S845A  MexE: S8F,A79G,A231T  MexF: A843T  MexX: A30T,K329Q,L331V,W358R  MexY: T543A,Q840E  NalC: G53E,S191R  AmpR: E109A,S174T,G278E,M283R  ftsI (PBP3): V537L  gyrA: T83I; parC: S87L,P752T; parE: D533E |

* Resistant determinants were identical in all three isolates sequentially obtained from the patient

ST-357 is among the top-10 international high-risk clones
